# Development of an Assistive Technology for Cognition to Support Meal Preparation in Severe Traumatic Brain Injury: User-Centered Design Study

**DOI:** 10.2196/34821

**Published:** 2022-08-04

**Authors:** Stéphanie Pinard, Carolina Bottari, Catherine Laliberté, Hélène Pigot, Marisnel Olivares, Mélanie Couture, Aline Aboujaoudé, Sylvain Giroux, Nathalie Bier

**Affiliations:** 1 École de réadaptation Université de Montréal Montréal, QC Canada; 2 Centre de réadaptation Estrie CIUSSS de l'Estrie-CHUS Sherbrooke, QC Canada; 3 Centre de recherche interdisciplinaire en réadaptation du Montréal Métropolitain Institut universitaire sur la réadaptation en déficience physique de Montréal CIUSSS Centre-Sud-de-l'Île de Montréal Montréal, QC Canada; 4 DOMUS Laboratory Department of Computer Science Université de Sherbrooke Sherbrooke, QC Canada; 5 Centre de recherche sur le vieillissement CIUSSS de l'Estrie-CHUS Sherbrooke, QC Canada; 6 Centre for Research and Expertise in Social Gerontology CIUSSS West-Central-of-Montreal Côte Saint-Luc, QC Canada; 7 Department of Psychology Université de Sherbooke Sherbrooke, QC Canada; 8 Research Center Institut universitaire de gériatrie de Montréal CIUSSS Centre-Sud-de-l'Île de Montréal Montréal, QC Canada

**Keywords:** user-centered design, needs assessment, assistive technology, brain injury, activities of daily living, cognitive rehabilitation, meal preparation, mobile phone

## Abstract

**Background:**

Although assistive technology for cognition (ATC) has enormous potential to help individuals who have sustained a severe traumatic brain injury (TBI) prepare meals safely, no ATC has yet been developed to assist in this activity for this specific population.

**Objective:**

This study aims to conduct a needs analysis as a first step in the design of an ATC to support safe and independent meal preparation for persons with severe TBI. This included identifying cooking-related risks to depict future users’ profiles and establishing the clinical requirements of the ATC.

**Methods:**

In a user-centered design study, the needs of 3 future users were evaluated in their real-world environments (supported-living residence) using an ecological assessment of everyday activities, a review of their medical files, a complete neuropsychological test battery, individual interviews, observational field notes, and log journals with the residents, their families, and other stakeholders from the residence (eg, staff and health professionals). The needs analysis was guided by the Disability Creation Process framework.

**Results:**

The results showed that many issues had to be considered for the development of the ATC for the 3 residents and other eventual users, including cognitive issues such as distractibility and difficulty remembering information over a short period of time and important safety issues, such as potential food poisoning and risk of fire. This led to the identification of 2 main clinical requirements for the ATC: providing cognitive support based on evidence-based cognitive rehabilitation to facilitate meal preparation and ensuring safety at each step of the meal preparation task.

**Conclusions:**

This needs analysis identified the main requirements for an ATC designed to support meal preparation for persons with severe TBI. Future research will focus on implementing the ATC in the residence and evaluating its usability.

## Introduction

### Background

Assistive technology for cognition (ATC), which are devices and software designed to meet the specific needs of persons with cognitive deficits, hold great promise [1-10]. However, few have been designed based on an exhaustive understanding of the complex and unique needs of individuals who have sustained a severe traumatic brain injury (TBI). TBI is defined as an alteration in brain function caused by an external force, such as a car accident, causing cognitive, physical, behavioral, and emotional disabilities [11]. These disabilities have an important impact on engagement in Activities of Daily Living (ADL) [12], and as most TBI survivors are young adults, they will live for an average of 50 years with the resulting difficulties [11]. When considering the extremely high lifetime care costs associated with severe TBI [13], providing safe and adapted environments, including ATC that enable the functioning of TBI survivors, should be deemed a societal priority.

ATC can help individuals with TBI realize their domestic and community activities [14,15]. In a recent meta-analysis, Nam and Kim [16] concluded that assistive devices may be an effective intervention for people with brain injuries. In addition, individuals living with moderate to severe TBI and their caregivers have expressed an interest in and willingness to use ATC [1,17]. Although a wide range of potentially supportive ATC exists, few have been developed with the active participation of persons with TBI and their families. Hence, their design may not capture the complexity of the cognitive needs associated with brain injury or the factors contributing to their acceptance and adoption in real-life settings. In addition, the design of most over-the-counter technological devices does not target the specific needs of persons with TBI, making it generally challenging for this population to use these devices independently.

To meet the needs of people with TBI and create useful and effective ATCs, our team developed a partnership with a supported-living residence for TBI in the province of Quebec, Canada. All stakeholders, including residents with TBI, actively participated in setting up a living laboratory to implement innovative technologies. The residence accommodated 10 people with severe cognitive deficits but negligible physical impairments, requiring 24/7 staff supervision. Of these 10 people, 6 (60%) lived in small apartments with cooking facilities, and 4 (40%) lived in basic rooms. All residents had access to common areas, including a central cafeteria where staff served daily meals. The residence was associated with a regional rehabilitation center.

In 2013, we completed the first study, with 7 residents, 4 caregivers, and 5 health care providers working at the residence. The goal was to identify and rank daily needs that they would like future ATCs, that would be developed by our team, to address in the context of a living laboratory project designed to support the specific needs of all stakeholders from this particular residence [18]. Meal preparation was identified as a priority [18]. At the time, no resident had permission to cook with a stove because of the high level of risk involved (eg, fires and burns). Residents were only allowed to prepare light meals, such as breakfast. In addition, to the best of our knowledge, no ATC was commercially available at that time to support meal preparation by persons with TBI. Although descriptions of 3 prototypes had been published in peer-reviewed journals [19-22], they could not be used in the context of this project, as they had not been researched, designed, or adapted to the needs of individuals with TBI. For instance, the first technology used a robot and did not provide assistance adapted to the needs of persons with TBI [19]. The second was a cooking support system that used kitchen sensors and displayed cooking instruction videos. Although this system provided assistance adapted to the user’s progress [21], it was not designed for the specific needs of people living with TBI. The third [22] was an application called Smart Kitchen for Ambient Assisted Living, tested for older adults. This application showed good usability and cognitive accessibility, but it was neither specific to TBI nor designed considering evidence-based cognitive rehabilitation practices. More recently, Wang et al [21] published a feasibility study of an automatic, context-aware, prompting system designed to support persons with TBI with multitasking specific to cooking. This study provided a starting point for the potential of ATC for people with TBI during meal preparation. However, although helpful, the ATC’s design is limited to guiding the person step by step through task performance. Though this type of compensatory approach is well supported for persons living with TBI, it fails to grasp the full potential of ATC, as it does not consider the breadth of other possible rehabilitation approaches such as metacognitive strategies considered as evidence-based rehabilitation strategies in TBI [23].

In the context of this study, which was conducted using a living laboratory approach [24], we aimed to co-develop with, and for, the residents with TBI, an ATC that would support their needs to prepare meals safely but also tap into their rehabilitation potential by implementing evidence-based cognitive rehabilitation interventions to optimize their independence in meal preparation. To do so, we used a user-centered design (UCD) method involving the following steps: (1) needs analysis, (2) design and prototyping, (3) experimentation, and (4) iterative follow-up [25-27]. Research on ATC development has shown the importance of considering the user, including persons with TBI, at all stages of the UCD process [28-31]. The continuous involvement of future users leads to the development of safer, more effective, and efficient products and enables faster postdesign deployment [29], smoother transfer into the environment [31,32], contributing to product acceptance and overall future success [29,33]. Residents with TBI were thus considered as equal members of the design team throughout the design process.

### Objectives

The general goal of this project was to conduct a needs analysis of the residents and develop an ATC for meal preparation as requested by them. More specifically, the study aimed to (1) depict future users’ profiles, including their difficulties in meal preparation; and (2) establish the clinical requirements for designing an ATC that would support meal preparation accordingly. The subsequent steps of the project were to design the ATC, implement it in the residence, and explore its usability. These steps have been previously published elsewhere [34]. The ATC was ultimately named the *Cognitive Orthosis for Cooking (COOK)*.

## Methods

### Overview

As mentioned earlier, we used a UCD method to collect data pertaining to the needs analysis step of an ATC design. To do so, this study was separated into two parts based on two specific objectives: (1) methods used to depict the future users’ profiles and (2) methods used to determine the clinical requirements for the ATC. The needs analysis was conducted over a 24-month period between July 2014 and August 2016.

### Conceptual Framework Supporting the Study

We opted to use the Disability Creation Process [35] as a conceptual framework. This framework is used in all rehabilitation centers in Quebec, including the supported residence where this study took place. It allowed for a shared vocabulary among stakeholders, which is very important in a living laboratory involving multiple stakeholders, and was a facilitator both for collaboration [36] and for the conceptualization of the ATC’s requirements.

According to the Disability Creation Process, a person with TBI experiences a disabling situation, which has the potential to be modified to facilitate more complete social participation. Full social participation refers to *the total accomplishment of life habits, resulting from the interaction between personal factors (impairments, disabilities, and other personal characteristics) and environmental factors (physical or social; facilitators and obstacles).* Life habits are defined as regular activities (eg, eating meals, communicating with others, and moving around) and social roles (such as holding a job) that ensure a person’s survival and well-being in society [35]. When a person can achieve full social participation, they are considered independent [37]. On the other end of the spectrum of social participation, there is a disabling situation, which is defined as “the reduced accomplishment of life habits, resulting from the interaction between personal factors and environmental factors.” In a disabling situation, a person is considered dependent on others to complete a given task.

In this study, the framework was used to help determine how the ATC could support the independence of a person with TBI in terms of features and services; or, more precisely, what were the *clinical requirements* that had to be addressed by the ATC. Identifying these requirements is a prerequisite for the design of any ATC [38]. In accordance with the framework, they should address all components leading to disability in meal preparation: (1) addressing the identified impairments (eg, rehabilitation of executive functions), (2) providing environmental compensation for the person’s deficits in the event of a dangerous situation (eg, cutting the stove’s power supply), and (3) simplifying the activity (eg, guidance for the preparation of a simple meal using a step-by-step format that is easy to follow).

### Participants

Out of the 10 resident members of our living laboratory, 6 (60%) could participate in the development of the ATC, as they lived in small apartments with cooking facilities. The other 40% (4/10) lived in basic rooms. The selection criteria to participate in this study were as follows: (1) to be motivated to participate in the study, (2) to present a stable life situation (eg, not currently experiencing a period of heavy alcohol consumption or major life stressors), and (3) to demonstrate potential to resume meal preparation as evaluated by the rehabilitation team working at the residence. The exclusion criteria consisted of a diagnosis of depression or any other significant medical condition that could impede participation in the study. Of the 6 participants, 3 (50%) met the inclusion criteria and are identified in the text as resident 1, resident 2, and resident 3. Although the ultimate objective of our team was to develop an ATC that would be useful for diverse profiles of persons who have sustained a TBI, we had to start with the specific needs of these 3 residents, with the intention of progressively increasing the number of functionalities in the future. The other stakeholders (2 caregivers, 3 residence staff, 3 health care professionals, and the 2 administrators of the residence) also agreed to participate in the project.

### Ethics Approval

This study was approved by the Ethical Review Board of the Centre for Interdisciplinary Research in Rehabilitation of Greater Montreal (reference CRIR-19-11-2013) and the Ethical Review Board of the Centre Intégré Universitaire en Santé et Services Sociaux De L’Estrie-Centre Hospitalier Universitaire De Sherbrooke (reference 2017-715-IUGS). Procedures followed by the ethical review boards were in accordance with the ethical standards of committees responsible for human experimentation in Canada and in the province of Quebec. All participants and their legal guardians, when required for residents with severe TBI, gave their written informed consent.

### Part 1: Depict Future Users’ Profiles—Data Collection for Objective 1

Table 1 presents the data collection tools that were used to determine the users’ profiles. A detailed portrait of the challenges specific to the 3 future users was prepared based on the Disability Creation Process [35], including an evaluation of personal factors, life habits (regarding meal preparation), and the environment. The process was led by 2 occupational therapists (SP and CL). To document personal factors, residents’ medical files were reviewed (including medical reports and physiotherapy and occupational therapy reports), and each was administered a complete neuropsychological test battery. This battery comprised the following tests: Trail Making Test A and B [39], Wechsler Adult Intelligence Scale 4th Edition (Digit Span Forward, Letter-Number Sequencing, Digit Span Backward, Block Design Visuospatial and Motor Skills, and Visual Logic Reasoning [40]), Rey Auditory Verbal Learning Test (word list) [41], Brief Visuospatial Memory Test-Revised [42], Delis–Kaplan Executive Function System Color Word Interference Test (Stroop) [43], and the Tower of London Mental Flexibility-Drexel University [44].

Residents were also interviewed regarding their perception of using technology to support meal preparation, their expectations of the future ATC, and their personal objectives and expectations related to resuming meal preparation activities.

To document life habits, a team of occupational therapists (SP, CB, and NB) led the process of documenting each resident’s profile. Four data sets were collected: independence in everyday activities before the TBI, current level of independence in meal preparation at the residence, number of light meals prepared per week without using a stove, and level of independence and satisfaction with all life habits.

**Table 1:** Data collection to depict future users’ profiles.

Categories and data sets			Tools
**Personal factors**			
	Medical files		Hand search
	Neuropsychological assessment of the 3 residents		Complete neuropsychological test battery
	Perception of technology		Individual interviews with the participants
**Life habits**			
	Independence in everyday activities before the TBI^a^		ADL^b^ Profile [45,46]: individual interviews with the participants and their family members
	Independence in meal preparation at the residence		IADL^c^ Profile [47-50]: performance-based assessment; ADL Profile questionnaire [45,46] with the participants and their family members
	Number of meals prepared per week		Observation log journal kept by the residence staff to document the number of meals prepared
**Environment**			
	Obstacles or facilitators to meal preparation		Field interviews and observations in situ

^a^TBI: traumatic brain injury.

^b^ADL: Activities of Daily Living.

^c^IADL: Instrumental Activities of Daily Living.

To assess the level of functioning of participants before their TBI and at present, we conducted a review of their medical records, individual interviews (based on the ADL Profile) [45,46], and interviews with a family member. Current level of independence in meal preparation was assessed with the Instrumental Activities of Daily Living (IADL) Profile [47], a performance-based measure of independence in IADL viewed through the lens of executive functions. This assessment used a nondirective approach and was administered in the person’s home and community environment. The IADL Profile consists of 3 scenarios (inviting someone for dinner, obtaining information, and making an annual budget) that the person is invited to think through and carry out in their home and community environment. Considering the focus of the research project, we only completed the first scenario, which included six interrelated tasks: (1) putting on outdoor clothes, (2) going to the grocery store, (3) shopping for groceries, (4) preparing a hot meal, (5) having a meal with a guest, and (6) cleaning up after the meal. The tool has excellent psychometric qualities and has been extensively validated with individuals who have sustained a moderate or severe TBI [48-50]. For 2 residents, the meal preparation was videotaped to enable a more detailed analysis of their performance and identification of any at-risk behaviors. For the third resident, extensive notes were taken during the evaluation.

The IADL Profile was administered to each participant 3 times, in part or in full, depending on their level of collaboration. Slight variations were made to the tool’s standard instructions when administering the tool for the second and third times. These variations were used to allow for an observation of different potential contexts of use of the technology and the associated performance of future users. Three meals were prepared by resident 1 (simple spaghetti, hot sandwiches and cookies, and meat loaf) and resident 3 (minestrone soup, roast beef and vegetable rice, and sauerkraut and sausage), and only 2 by resident 2 (meat macaroni and a chicken Caesar salad) because of his limited cooperation.

The number of meals prepared by the participants each week was documented in an observation log. The log was completed using a short daily interview (conducted by CL) with the residence staff. It consisted of a chart created to document tasks completed over 5 consecutive days, collecting the number of meals prepared by each participant and including whether it was a cold or hot meal. The log also allowed us to record each participant’s failures and successes in unsupervised meal preparation.

Each resident’s social and physical environments were also documented with field observations by residence staff as well as by formal and informal discussions between members of the research team and all stakeholders. The stakeholders included the rehabilitation team (ie, social workers, specialized education technicians, and nursing staff), residence staff, and managers. Information was collected using written memos during informal interviews and formal meetings about the project.

### Data Analysis for Part 1

A deductive qualitative analysis [51] based on the Disability Creation Process was used to organize the data collected from multiple sources and yielded the level of social participation in meal preparation for each participant. The data were analyzed by SP, and the content was validated by CL, CB, and NB. Discordance was discussed among all evaluators, and a consensus was reached for all information classified in the Disability Creation Process. Results of each IADL assessment were validated by SP, CL, and one of the authors of the IADL Profile assessment (CB) to increase the validity of the results.

### Part 2: Establish the Clinical Requirements for the Assistive Technology

The most relevant interventions that could be offered to address the needs of future users were identified, and the recommended clinical interventions were translated into requirements to guide the ATC design. The following steps were used to translate user needs into clinical requirements for the ATC: (1) identify evidence-based practices known to improve independence in persons with TBI, (2) from among these practices select those that are appropriate to the needs of each future user and are most likely to improve their independence (individualized intervention plan), and (3) identify the clinical requirements for the future ATC for meal preparation to guide design and technological development.

To identify evidence-based practices known to improve independence in persons with TBI, a rapid review was conducted by clinical specialists on the design team. This team of clinical specialists included 4 occupational therapists: a doctoral student in rehabilitation sciences (SP), 2 academics and clinical scientists (CB and NB), and a research coordinator (CL). Cognitive rehabilitation can be defined as systematic therapeutic activities that aim to improve injury-related deficits to maximize safety, daily functioning, independence, and quality of life [23]. Although numerous evidence-based clinical recommendations for the cognitive rehabilitation of persons with TBI have been published [23,52-54], it is those of Haskins et al [23] and Bayley et al [53] that were adopted in this study. For Haskins el al [23], this choice was based, in part, on the support from the American Congress of Rehabilitation Medicine and the accompanying practice guidelines that assist in their application. Regarding the Guidelines for Cognitive Rehabilitation following TBI proposed by Bayley et al [53], the choice was made because of its rigorous development process, including an extensive literature review.

Each resident’s intervention plan was developed according to (1) an analysis of the interaction of the resident with his occupation and living environment, combined with his individual needs to promote engagement; and (2) evidence-based cognitive rehabilitation interventions found in the rapid review.

Finally, to identify the clinical requirements for the future ATC, the research team *translated* each intervention plan into usable terms for the computer science team (eg, the ATC should be automatically shut down if a burner is left open on the stove for an extended period of time). To this end, team members listed the difficulties observed during meal preparation for each of the 3 participants and added complementary information obtained from the stakeholders. Subsequently, a list of possible functionalities of the ATC was defined (eg, to support meal preparation with or without recipes, to support grocery list preparation, and to support budget management related to shopping for meal preparation). A classification of the level of importance for each functionality was established by the design team according to whether the functionality was considered necessary, ideal, or optional for each participant.

## Results

### Future Users’ Profiles

Participants’ social participation in meal preparation was analyzed according to the Disability Creation Process. The entire process included up to 6 in-person meetings with each of the future users, 2 meetings with resident 1’s mother, 1 meeting with resident 2’s mother, and up to 6 meetings per resident with the residence’s staff, health care professionals, and administrators.

### Personal Factors

The complete profile of each participant is presented in Table 2. All 3 participants were single, middle-aged men with physical and cognitive disabilities. They could stand up and walk with (resident 1) or without (resident 2 and resident 3) an orthosis and could use both hands to at least stabilize objects (resident 1). Resident 1 had a left hemiparesis. Resident 3 presented with anosmia, deafness, and severe food allergies. Cognitive impairments in residents 1, 2, and 3 could interfere with meal preparation tasks and have an impact on safety. These included deficits in working memory and executive function (residents 1, 2, and 3), episodic memory (resident 1), and abstraction and reading difficulties (resident 1 and resident 2). All participants were able to name some of their cognitive impairments but not their impact on their performance in a meal preparation task.

**Table 2:** Residents’ profile and personal factors.

Personal factors			
Participant	Medical file	Neuropsychology analysis	Perceptions and expectations about the ATC^a^
Resident 1	Male, aged 48 years Severe TBI^b^, 19 years since TBI 11 years of education Hemiparesis to his left hemi-body	Mild difficulties related to short-term memory and working memory Mild difficulties in reasoning and difficulties in problem-solving (planning) Anxiety, impulsivity, and behavioral outbursts	Perceptions: open to using technology but anxious about his ability to learn to use technology Frequently uses his computer for social networking and to surf on the internet Expectations To cook his own sauces with alcohol as before To cook a spaghetti sauce To prepare all his meals Motivated and collaborative
Resident 2	Male, aged 37 years Severe TBI, 32 years since TBI 9 years of education Chronic pain in the feet and back and chronic headaches	Mild deficits in working memory Difficulty alternating between 2 concepts; mild difficulties in reasoning Difficulty following verbal commands, reading, and calculating quantities	Perceptions: open to using the ATC but anxious. Says that he will need help Frequently uses his own computer for social networking Expectations To eat what he wants when he wants To prepare a recipe for bœuf bourguignon de la France To manage his budget and grocery list with assistance Agreed to participate in the project but said that he does not need help to cook
Resident 3	Male, aged 55 years Severe TBI, 10 years since TBI 15 years of education Several food allergies Deafness, lack of dexterity with his right hand, and balance problems	Very slow processing visual information Difficulty alternating between 2 concepts but able to plan and solve problems in some contexts Difficulty with episodic memory with no improvement when the material is repeated and loss of the information after a delay	Perception: open to using the ATC and not anxious because he had used technology in his work before his TBI Frequently uses his computer to search for information on the internet Expectations To have the possibility of eating alone in his apartment To cook simple meals (soup) for his evening snacks To be able to prepare pasta Generally collaborative, but this varied over time

^a^ATC: assistive technology for cognition.

^b^TBI: traumatic brain injury.

### Life Habits

#### Overview

Before their TBIs, resident 1 and resident 3 were completely independent in managing their life habits and social roles, including cooking. Resident 1 used to be a chef. Resident 2 had his TBI at the age of 5 years. Independence in meal preparation after his TBI at the residence is presented in the following sections.

Overall, the 3 residents were dependent on others for carrying out at least one life habit and were under a trusteeship to manage their finances. All 3 required 24-hour supervision owing to the high level of verbal assistance needed to facilitate their functioning and to ensure their safety. All of them relied on cafeteria services for their meals. None had permission to use a stove, and all were dissatisfied with their functioning in meal preparation. The detailed profile of each resident is presented in the following section.

#### Resident 1—Independence in Meal Preparation at the Residence

Before his TBI, resident 1 worked as a cook in restaurants and was, therefore, able to prepare complex meals. He enjoyed creating new healthy recipes for himself. At the onset of the project, resident 1 generally ate a simple breakfast (eg, a muffin) in his apartment and all other meals at the cafeteria. Hence, for the last 20 years, resident 1 had not cooked any meals, except for the few times when his cooking ability was assessed in rehabilitation or to help a friend during a visit.

In general, based on the IADL Profile, resident 1 was able to independently formulate a goal, plan, and carry out a well-known simple meal preparation using the stove, without a recipe, and verify the attainment of the goal. However, he needed verbal assistance to carry out a new recipe that was given to him. For example, he had to reread certain parts of the recipe 4 to 5 times to remember the cooking time and temperature required for baking cookies; he still made mistakes with both. Potential risks were identified, such as forgetting something on the burner while consulting social media, eating raw beef, handling a hot plate in a dangerous manner, using the stove as a place to store plates but forgetting to remove them when turning on the stove, and carrying boiling water in an unsafe manner. In addition, he was exhausted at the end of the evaluation. The major issues for him were, therefore, distractibility, energy management, and difficulty in remembering information over a short period of time (ie, remembering the cooking time until he programed the timer). His level of fatigue also had an impact on his level of anxiety, an observation that was confirmed by the evaluation as well as by the staff and resident 1’s mother.

An important element was reported by the mother. She told the evaluator that the evaluations seemed to have had a very positive impact on his self-awareness and on his functioning in general. She told the evaluator, “I don’t know what you did with him, but please continue. He has never been so aware of his difficulties in the last 20 years” (note from memos). Resident 1 also demonstrated a capacity to learn. After the first IADL Profile assessment, he received feedback on safety issues; he then modified all his behaviors accordingly during the second evaluation.

Resident 1 prepared an average of 7 simple meals per week in his apartment, preparing only breakfast (eg, toast or muffin with coffee) with no stove access.

#### Resident 2—Independence in Meal Preparation at the Residence

Resident 2 had sporadically worked for a few hours per week as an assistant cook in different restaurants. He mentioned difficulties when preparing meals, such as forgetting to turn off the tap or burner if he was distracted at work. Before being involved in the study, resident 2 ate most of his meals at the residence’s cafeteria but frequently ordered fast food from the restaurant, although he was struggling financially. He frequently ate the same type of food. He cooked easy meals that did not require him to follow recipes (eg, macaroni) in a microwave oven or on an electric cooking plate (discreetly and illegally) in his apartment.

The IADL Profile was very difficult to administer to this participant, and the evaluator had to make major modifications to the presentation of the evaluation because of resident 2’s behavioral problems. He cooperated during the first evaluation, although he needed assistance in choosing the recipe and did not want to be videotaped. However, he was able to prepare the meal (ie, macaroni with meat and vegetables) without difficulty or any safety issues. The second evaluation was more difficult to administer, because he refused to use the oven to cook and made a Caesar salad with baked chicken, for which the evaluator (SP) had to provide a considerable amount of assistance in formulating the goal and planning. He was able to carry out the task and verify the attainment of the goal by himself. He refused to undergo a third evaluation. To complete the assessment, the evaluator had to change the evaluation approach toward a more collaborative one by suggesting that they *make a meal together*. During this meal preparation involving the use of a recipe, he had difficulty reading and understanding the information as well as calculating the quantities. Therefore, he needed a considerable amount of verbal assistance to carry out the meal. During the evaluation, the evaluator noted a lack of hygiene: he did not wash his hands after manipulating the cat litter while he was cooking and was not motivated to clean up after meal preparation; upon the evaluator’s insistence, he asked for help in cleaning and said that he did not care about cleanliness. During all 3 meal preparations, the main safety issues noted for resident 2 were the risk of food poisoning because of hygiene issues that did not appear to bother him (eg, not cleaning before and after cooking, manipulating food and cat litter at the same time, and not cleaning up dead flies and dirty dishes), the risk of falling because of the presence of a cat, the risk of fire owing to forgetting something on the stove while stepping outside to smoke, and the poor organization of his apartment (paper and objects lying around, and on the stove).

The staff and rehabilitation professionals also noted issues related to perseveration and hygiene. According to the observations made by his health professionals, resident 2 had difficulty diversifying his menu over a 1-week period and tended to repeatedly eat the same foods (eg, he ate Caesar salad every day for a whole month). They also reported that he had difficulty cleaning his apartment, more precisely in initiating the activity and required prompts to do so. The staff also identified safety issues related to cooking, because resident 2 was cooking food with a propane camping stove in his apartment (it was removed from the apartment when the staff became aware of it).

#### Resident 3—Independence in Meal Preparation at the Residence

Resident 3 mentioned being a good cook before his TBI through following recipes. He avoided restaurants because of his severe food allergies. Since his TBI, he has never had the occasion to cook again.

During the first IADL Profile evaluation, he formulated the goal of preparing a meal independently and decided to prepare a simple minestrone soup following a recipe in a cookbook. During subsequent evaluations, we observed that he functioned better with a recipe than without, because he did not have to improvise. He prepared his shopping list independently, based on the ingredients in the cookbook. He also used his list adequately when at the grocery store. However, he was dependent on the evaluator to verify whether the ingredients were safe for him to eat, considering his allergies. In fact, he twice bought ingredients that were dangerous for him, and planned to eat them anyway, despite extensive cautionary verbal guidance from the evaluator. He was unable to adequately self-evaluate the goal attainment for preparing a meal, despite extensive verbal assistance. Moreover, he consistently said that he had adequately attained his goal even if the final meal was not of good quality and did not meet the initial task instructions (ie, inviting a guest for a meal). He served only broth to his guest and went to the cafeteria to eat instead of eating the meal he had prepared for himself and his guest. Other safety issues were noted regarding improper use of the stove (difficulty using the controls properly) and lack of hand hygiene before and during cooking. He also mentioned his concern about not being able to smell burning food because of anosmia. Resident 3 did not cook at all in his apartment.

Residence and rehabilitation staff were worried about his inability to manage his allergies. An incident of mismanagement of his allergies once sent him to the emergency room, despite very attentive and cautious cafeteria services. Therefore, he was considered dependent on another person to buy food that contained none of his allergens.

### Environments

All 3 residents lived alone in a 3 and a half apartment at the supported-living residence. Each apartment had an open-concept floor plan for the kitchen and living room, a bedroom, and a private bathroom. Possession and use of standard stoves were prohibited for safety reasons. Each apartment was equipped with 3 emergency call bells, and cafeteria services (3 meals per day) were available in the building. The social environment of these 3 participants included (1) caregivers (resident 1: mother, resident 2: mother, and resident 3: none); (2) residence staff who were on site 24/7 to provide supervision and support; (3) health professionals employed by the rehabilitation center affiliated with the residence, who carried out intervention plans; (4) residence manager, who managed staff and the logistics of the residence; and (5) coordinator of the research projects’ clinical team, who was trained in occupational therapy.

### Clinical Requirements for Designing the ATC

From evidence-based practice guidelines in TBI [23,53], the team identified six types of approaches for cognitive interventions: (1) compensating for the cognitive deficits with external aids (eg, using a calendar or smartphone to manage a schedule), (2) modifying environmental factors (eg, turning the television off when engaging in a complex task such as cooking), (3) incorporating strategies to promote generalizations by increasing the metacognition of the person with regard to his difficulties and ability to find solutions and providing education, (4) task-specific training to engage the person in meaningful activity in their own environment, (5) metacognitive strategy training (eg, Cognitive Orientation to Occupational Performance [55] or multicontext approaches [56,57]), and (6) restorative treatment such as training to address specific cognitive deficits (eg, training attentional capacities).

For the ATC design, the team selected three of these evidence-based intervention approaches based on the difficulties identified in the 3 participants [23]: (1) task-specific training to facilitate the learning of new routines in meal preparation, (2) compensation interventions or external strategies to compensate for cognitive impairments, and (3) metacognitive strategy training (specific to meal preparation or otherwise). These approaches were in line with the team’s intention to develop an ATC with both restorative and compensatory functions. Table 3 presents the interventions selected to address and provide support for the residents’ difficulties.

Clinical requirements for promoting safety and limiting the impact of cognitive impairments are presented in Tables 4 and 5. The goals chosen for the ATC were to support independence, functioning, and safety during a meal preparation task. Supporting *independence* means that the ATC must allow residents to cook in their residences independently, safely, and without human assistance. Supporting *functioning* means that the ATC must support the person during the actual meal preparation. Supporting *safety* means that the ATC must ensure not only the safety of the participants within their individual apartments but also that of the residence where several other people with cognitive impairments also live.

**Table 3:** Cognitive intervention plan for each participant.

Participant and main challenges interfering with meal preparation		Approaches	Specific interventions
**Resident 1**			
	Impaired awareness Fatigability and anxiety Distractibility Working memory deficits Forgetting to plan side dish Difficulty following recipes Unsafe behavior	Increasing awareness Metacognition	Video feedback [58]: identifying the behaviors that need to be modified COOP^a^ global strategy [55] Energy management: identifying more demanding activities Schedule management: avoiding planning to do 2 tasks at the same time to facilitate energy management Time pressure management [59] Pacing [60]
		Education	Training on safety issues surrounding cooking: increasing level of knowledge about safety to help change behavior
		Task-specific compensation	Logbook [60]: writing down any ideas or concerns not related to the cooking task to avoid internal distractors Stop and think [23]: a stop sign as a reminder to concentrate on the cooking task Reminders to modify behavior before and during the task: (eg, do not eat raw meat and check oven before cooking) Checklist to integrate better habits; for example, check before cooking that your Facebook and phone are turned off and the sign on the door is in place (do not disturb) Adaptation (recipe presented on a single page, highlight vital information, etc) and repetition of recipes (spaghetti sauce recipe) Human assistance for grocery shopping and budget management
**Resident 2**			
	Abstraction and attention difficulties Safety behavior Difficulty following recipes Apartment-cleaning issues Difficulty preparing a balanced meal plan for the week that includes healthy choices and not eating the same thing every day	Task-specific compensation	Integration of a routine to clean before and after the task with checklist, reminders, and human assistance Support in developing a weekly meal plan: schedule, list of healthy meals selected with him, and human assistance to plan Positive behavior reinforcement regarding cleaning Adaptation of the recipe and repetition of recipes important for him Human assistance for grocery and budget management
		Education	Training on safety issues related to cooking: increasing level of knowledge about safety to modify his behavior
**Resident 3**			
	Allergy management Difficulty with his selective attention	Task-specific compensation	Reminders and human assistance when purchasing ingredients at the grocery store and follow-up home verification of potential allergens before cooking Adaptation of recipes to facilitate meal preparation
		Education	Training on safety issues related to cooking: increasing level of knowledge about safety to modify his behavior

^a^COOP: Cognitive Orientation to Occupational Performance.

**Table 4:** Translation of security needs into clinical requirements.

Safety needs and clinical requirements^a^			Prioritization^b^
**Decrease risk of fire or injury if stove left unattended during cooking**			
	*1 and 2—verbal and visual assistance* (prompting): ask user to watch what is on the stove when needed (*at the right moment; context-aware*)		2
	*3—Compensation*: the ATC^c^ must shut down the stove if the user steps away and does not return to watch what is on the burners		1
**Decrease risk of fire or injury if burner left turned on and forgotten**			
	*1 and 2—Verbal and visual assistance* (prompting): ask the user to turn off the burner		2
	*3—Compensation:* turn off the stove if the user does not turn off the burner		1
**Decrease risk of injury if oven door left open and forgotten**			
	*1 and 2—Verbal and visual assistance* (prompting): ask user to close the oven door		2
**Support routine about hygiene and cleanliness management**			
	*2—Verbal and visual assistance* (prompting): remind user about good hygiene habits (eg, wash hands before cooking)		2
**Support routine checking of expired food to prevent food poisoning**			
	*2—Verbal and visual assistance* (prompting): provide relevant information on expiry dates of prepared foods		3
**Decrease risks related to severe allergies**			
	*3—Compensation*: prevent user from cooking before ingredients are verified by an employee		1
	*2—Verbal and visual* assistance (prompting): remind user to check if he has his EpiPen (allergy emergency medication) before cooking		2
	*3—Compensation* (supervision): only allow the employee to reactivate the stove after the safety lock has been activated		1

^a^1: detect the problem, 2: warn or assist the person, and 3: compensate for the problem.

^b^1: essential, 2: ideal, and 3: optional.

^c^ATC: assistive technology for cognition.

**Table 5:** Translation of cognitive needs into clinical requirements.

Cognitive needs	Clinical requirements^a^	Prioritization^b^
Support planning (eg, choose recipe and diversify menu)	*2—verbal and visual assistance*: support planning process by asking directed (or orientated) questions	2
Support difficulties in carrying out the recipes (eg, errors)	*3—task and environment adaptation:* types of recipes and the way in which recipes are presented must be adapted. Adaptations such as, for example, different colored measuring cups must be available to support these difficulties	2
Reduce internal distractions	*2—provide logbook*: provide a logbook that the user can use to discard his “distracting” thoughts and ideas before and during the task	2
Reduce external distractions	*2—provide reminders* and contribute to *increased awareness*: remind user to reduce distractors before starting the task	1
Manage fatigability	*2—pacing*: support the user’s planning of required breaks during the task	2
Manage fatigability	*3—reminder*: remind and request that the user take a break at the right time during the task	3

^a^1: detect the problem, 2: warn or assist the person, and 3: compensate for the problem.

^b^1: essential, 2: ideal, and 3: optional.

For independence, it was determined that the ATC should increase the number of meals prepared over a 1-week period. For functioning, it was determined that the ATC had to reduce performance errors. For safety, it was necessary to reduce errors leading more specifically to safety issues. If errors could not be avoided with the support of the ATC, human intervention would be planned in advance and, in certain instances, given as a preventive measure to ensure safety (eg, checking for potential food allergies in the grocery bag).

The research team translated each safety and cognitive need into design specifications. For example, to decrease the risks of fire, the clinical requirements were that the ATC had to (1) detect when the stove was left unattended (the problem), (2) warn the person about the problem, and (3) compensate by turning off the stove if the person did not react to the warning. The team determined that assistance would be provided both verbally and visually. Once all requested functionalities had been listed, they were prioritized by the team so that the ATC would support an *Agile* development method [61] and, more specifically, use a feature-driven development method [62] that would address each future user’s needs one at a time, progressively adding specific features as needed. This iterative and incremental approach was used to guide the development of a series of functional prototypes.

## Discussion

### Principal Findings

This study presented a needs analysis as the first phase of designing an ATC, named COOK, to support independence and safety in meal preparation for individuals with severe TBI and living in a supported-living residence. Using a UCD method, the needs analysis included two steps: (1) identifying the future users’ profiles, including their difficulties with meal preparation and (2) identifying the clinical requirements for the design of an ATC to support meal preparation based on the risks and future users’ profiles. The results showed that many issues had to be considered for the development of COOK for the 3 residents, including cognitive issues such as distractibility and difficulty remembering information over a short period of time and important safety issues, such as potential food poisoning and risk of fire on the stove. These issues led to two main clinical requirements to be developed by the team: (1) providing cognitive support based on evidence-based cognitive rehabilitation to facilitate meal preparation and (2) ensuring safety at each step of the meal preparation task. Our results also showed that using multiple sources of data, including the perspectives of the multiple stakeholders involved, led to an in-depth needs analysis that considered all the difficulties faced by the 3 residents.

The cognitive profiles that were documented in this study were consistent with the most frequent ones following severe TBI, including distractibility, problem-solving difficulties, difficulty remembering information, fatigue, and behavior problems [63,64]. Hence, this first prototype of COOK is based on 3 cognitive profiles but nonetheless responds to the most frequently documented difficulties of the population with chronic severe TBI. The addition of more functionalities to make it useful for individuals with a broader range of cognitive difficulties was planned as a subsequent step to this study in the ongoing iterative and UCD of the ATC. Other recent work on COOK has expanded its validation by testing its suitability for use by other persons having sustained a TBI or living with neurodegenerative diseases. Results from these other studies have shown that COOK’s main functionalities are well suited for a broader group of individuals with TBI [65-68] as well as in the continuum from normal aging to early Alzheimer disease [69].

Evidence-based cognitive interventions included in COOK comprise 3 recognized rehabilitation approaches: task-specific training, compensation interventions, and metacognitive strategies [23]. Although other approaches exist, these are the most recommended in the field of cognitive rehabilitation for TBI [23,53], making COOK a technology that can provide cognitive support to a large number of persons with TBI. Integrating evidence-based interventions for a TBI clientele in the design of an ATC is an emergent design strategy that will improve future efficacy. In the specific context studied here, the ATC will be a new intervention option to facilitate resumption of meal preparation, so it is essential to explore evidence-based practice guidelines in designing it [2,23,53]. In this study, we addressed the limitations of other existing prototypes to support meal preparation, which only integrated a step-by-step approach into the ATC [21]. In the future, adding other metacognitive strategies and educational approaches to the design of this ATC will provide greater flexibility to clinical specialists who will then be able to adjust the technology to various and complex needs.

As for the elements related to safety, to our knowledge, this study is the first to document, with specific details, the safety elements related to meal preparation in TBI. The main safety issues were the risks of stove fire and food poisoning. Exploring the risks related to meal preparation in this study showed that this is a complex activity with many safety issues, and these risks are exacerbated by cognitive impairments [70]. Indeed, being safe at home requires a person to be able to identify potential risks and hazards when cooking, develop and implement problem-solving strategies when they occur [71], and then evaluate the results of the strategy put in place [72]. However, persons who have sustained a severe TBI have difficulty recognizing situations of risk and solving problems, which in turn compromises their safety at home [73]. For these reasons, high-risk situations specific to meal preparation identified in this study (eg, serious food allergies) may not be fully addressed by technology and may still require human assistance to ensure safe meal preparation. This study also showed that meal preparation includes related tasks (eg, grocery shopping and budget management), which require the implementation of complementary interventions to the ATC to facilitate greater social participation. To our knowledge, this is the first time that an ATC was developed considering not only the support for one particular activity but also for a wide range of other elements, including other closely related activities. This study illustrates the importance of considering the complex interactions between personal factors, environments, and life habits, when developing and using an ATC, especially when the activity is complex and poses a high risk for a person’s safety.

### Strengths and Limitations

This study has several strengths. First, multiple sources of data and stakeholder perspectives were used to identify needs related to meal preparation, which increases the validity and generalizability of the results [29]. In this needs analysis process, the IADL Profile evaluation was found to add valuable information about the participants. Its nondirective approach provided a thorough understanding of the degree of independence that participants were able to sustain during a complex activity. The IADL Profile evaluation also helped to identify whether participants were able to find solutions and correct their errors related to meal preparation and safety issues, as well as what kind of assistance they needed. This evaluation is also congruent with the proposal by De Vito Dabbs et al [29] of completing a contextualized evaluation where the goal is simply to learn how users perform their tasks. De Vito Dabbs et al [29] proposed that the evaluator is the apprentice of sorts, and the user is the expert, which is in line with the underlying nondirective approach inherent to the administration of the IADL Profile. To our knowledge, our study is the first to detail the specific needs of persons with TBI in the context of a UCD study with such an evaluation.

Second, in this study, clinical professionals with an occupational therapy background led the needs analysis, and the design team perceived this to be an important strength, because it allowed for a detailed specification of the clinical requirements. Although completing a detailed and exhaustive evaluation of individuals with such severe injuries is time consuming, as it requires direct observation of performance, it is essential for the development of new technology for a proper understanding of the end user’s competence and needs.

Third, the use of an intervention plan as a means of facilitating communication between the clinical and technological teams facilitated the integrative synthesis essential to interdisciplinary work and clearly supported the exchanges between the supported-residence stakeholders and clinical and computer scientists collaborating on this project.

This study has some limitations that are important to consider. First, a limited number of housing resource residents participated in the design process. However, as noted earlier, the main cognitive profiles and cognitive rehabilitation approaches implemented in COOK are representative of the needs and clinical strategies that are most frequent in the TBI population. It is to be expected that the completion of more studies on COOK, with more persons with cognitive impairments, will improve the generalizability of the interventions provided by the ATC to different cognitive profiles. Second, the study was conducted in the specific context of a residence with supervised assistance provided 24/7. Thus, the results are only applicable in this specific context, as expected in a living laboratory project based on the needs of a specific group of stakeholders such as in our study. Future studies on COOK will need to determine whether these results can be applied to other living contexts such as persons with TBI living alone in their homes in the community or in other supported residences. Preliminary results indicate that COOK is also promising in these other contexts [65-68], although some modifications may be necessary to tackle their specificities such as the absence of 24/7 supervision.

### Conclusions

This study aimed to determine the design requirements for a new ATC, named COOK, to support meal preparation for persons with severe TBI. Here, we have reported the first steps of the development process. Results of the needs analysis showed that safety and cognitive support were the 2 main categories of needs that required an ATC. Evidence-based interventions were identified to guide the design of an ATC that can support these needs, using an empirically based foundation. This paper also proposed interesting tools to support interdisciplinary work to design an ATC, such as the use of a common framework and a detailed functional evaluation based on observation methods. The next step involved developing COOK and implementing it in the residence to evaluate and improve its usability [34] as well as validating its use with other persons with a wide variety of cognitive deficits and in different living contexts.

## Abbreviations

ADL: Activities of Daily Living
ATC: assistive technology for cognition
COOK: Cognitive Orthosis for Cooking
IADL: Instrumental Activities of Daily Living
TBI: traumatic brain injury
UCD: user-centered design
